# Massive Abdominal Wall Abscess in a Chronic Irreducible Epigastric Hernia Containing the Transverse Colon

**DOI:** 10.7759/cureus.108623

**Published:** 2026-05-11

**Authors:** Ioannis Katsarelas, Mohammad Husamieh, Athanasios Polychronidis, Konstantinos Popotis, Periklis Dimasis

**Affiliations:** 1 Department of Surgery, General Hospital of Katerini, Katerini, GRC; 2 Department of General Surgery, General Hospital of Katerini, Katerini, GRC

**Keywords:** abdominal wall abscess, abdominal wall surgery, emergency medicine, epigastric hernia, positron emission tomography computed tomography, surgery, transverse colon

## Abstract

Epigastric hernias typically contain preperitoneal fat, while the involvement of the transverse colon is an exceptionally rare clinical entity. We report a unique case of a 56-year-old patient presenting with a massive abdominal wall abscess complicating a long-standing, irreducible epigastric hernia. Clinical and radiological evaluation via computed tomography (CT) revealed that the chronic hernia sac contained the transverse colon, surrounded by an extensive fluid collection, without evidence of acute incarceration or frank bowel perforation. Emergency surgical intervention confirmed the presence of a viable transverse colon within the sac. The abscess was likely the result of chronic inflammation or subclinical micro-perforations. The procedure involved thorough abscess drainage, debridement, and successful reduction of the transverse colon back into the peritoneal cavity, followed by primary fascia repair. This case emphasizes that neglected, irreducible epigastric hernias can lead to severe infectious complications even in the absence of acute strangulation, necessitating a high index of clinical suspicion and prompt surgical intervention.

## Introduction

Epigastric hernias represent approximately 1.5% to 3% of all abdominal wall hernias and typically occur through a fascial defect in the linea alba, anywhere between the xiphoid process and the umbilicus [1]. Most epigastric hernias are small, often asymptomatic, and are frequently discovered during routine physical examinations as palpable, albeit non-tender, masses. In the vast majority of cases, the hernial sac contains only preperitoneal fat or omentum, while the involvement of hollow viscera is considered an exceptionally rare clinical entity, occurring in less than 5% of reported cases [2].

In the context of hernia complications, it is crucial to distinguish between incarceration and strangulation. An incarcerated hernia refers to a state where the herniated contents are irreducible due to adhesions or a narrow fascial neck, but their blood supply remains intact. Conversely, strangulation occurs when the vascular supply is compromised, leading to ischemia and eventual necrosis.

Although epigastric hernias are often small and easily manageable, neglected or long-standing cases can progress to severe complications. A particularly unusual and challenging presentation is the formation of a massive abdominal wall abscess, which can develop even in the absence of frank bowel perforation [3]. In these chronic cases, the inflammatory process is often the result of persistent pressure, chronic irritation, or subclinical micro-perforations of the herniated bowel wall, causing bacterial translocation into the hernia sac and leading to localized suppuration [4].

The diagnostic challenge in such instances lies in the clinical presentation, where the inflammatory signs of a massive abscess may mask the underlying hernia and its visceral content. A high index of clinical suspicion combined with early advanced imaging, such as computed tomography (CT), is vital for accurate diagnosis and surgical planning to prevent sepsis and further morbidity [4]. We report a rare case of a 56-year-old patient with a long-standing, chronically incarcerated epigastric hernia containing the transverse colon, which manifested as a massive abdominal wall abscess, necessitating urgent surgical intervention and debridement.

## Case presentation

A 56-year-old female patient presented to the Emergency Department with a painful, prominent mass in the epigastric region, accompanied by localized redness and warmth. The patient had a body mass index of 23.5 kg/m^2^, with no significant past medical or surgical history, and reported a known history of a chronic epigastric hernia for over five years, which had recently become symptomatic due to the underlying chronic incarceration. During the three days prior to admission, the mass became increasingly symptomatic, with intense localized pain and inflammatory skin changes. On presentation, the patient was febrile (38.2°C), while other vital signs were within normal limits (blood pressure: 130/85 mmHg, heart rate: 92 bpm). The patient denied other systemic or obstructive symptoms, such as chills, nausea, vomiting, or constipation.

**Figure 1:**
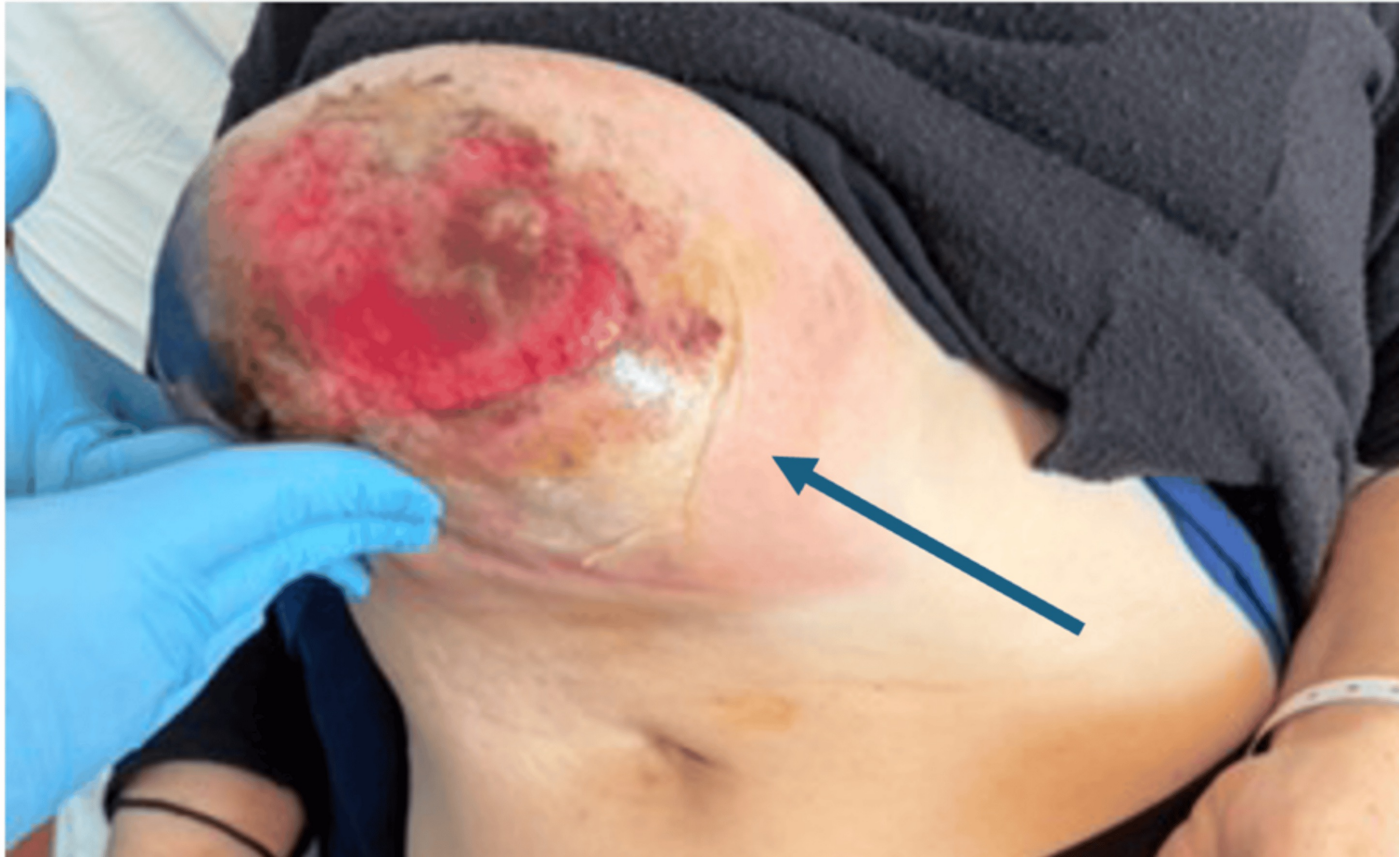
Clinical presentation of the abdominal wall abscess. Preoperative clinical photograph of the abdominal wall with a massive mass (blue arrow) in the epigastric region with intense inflammatory signs, suggestive of abdominal wall abscess.

Clinical examination revealed a large, firm, and exquisitely tender mass measuring 15 x 12 cm in the epigastrium. The overlying skin exhibited intense inflammatory signs, including necrotic changes and central fluctuance, highly suggestive of an extensive abdominal wall abscess (Figure 1).

Laboratory investigations on admission demonstrated a significant systemic inflammatory response and anemia, which was attributed to chronic inflammation. The anemia was managed conservatively and did not necessitate preoperative transfusion or delay of the surgical intervention. Key findings are summarized in Table 1.

**Table 1:** Key laboratory findings on admission.

Laboratory parameter	Patient value	Reference range	Units
White blood cell (WBC) count	17.5	4.0-11.0	×10³/μL
Neutrophils	88.2	40-75	%
C-reactive protein (CRP)	25.74	<0.5	mg/dL
Hemoglobin	10.8	12-16	g/dL
Hematocrit	34.1	36-47	%
Glucose	96	70-105	mg/dL
Serum urea	23	17-43	mg/dL
Creatinine	0.62	0.66-1.09	mg/dL
Sodium (Na^+^)	135	135-145	mEq/L
Potassium (K^+^)	3.74	3.5-5.1	mEq/L

**Figure 2:**
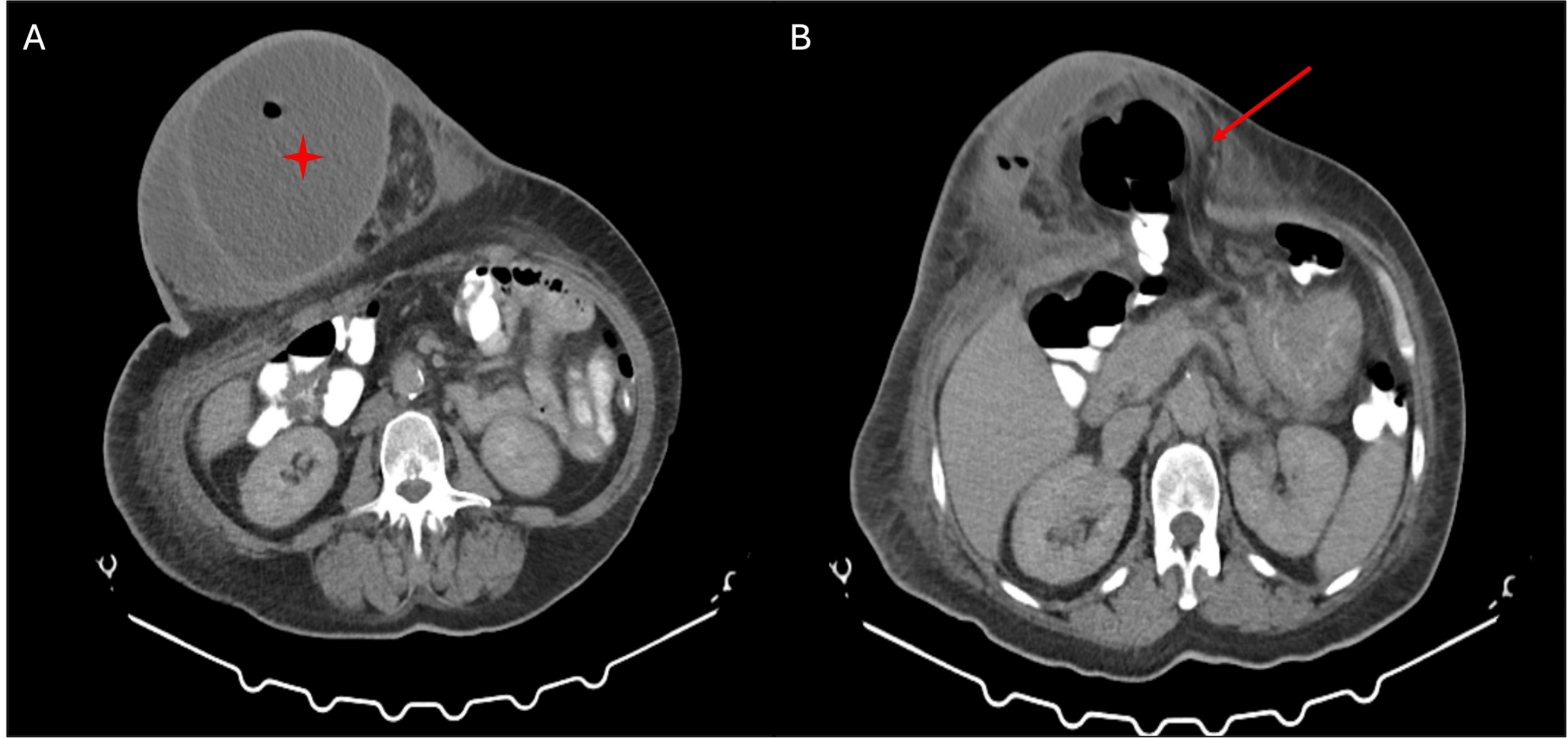
Contrast-enhanced computed tomography scan of the abdomen. (A) Axial view showing the extensive, multiloculated fluid collection and gas bubbles within the subcutaneous tissue of the epigastrium, consistent with a massive abscess (red star). (B) Axial view demonstrating a large fascial defect in the linea alba with herniation of the transverse colon (red arrow).

The patient was admitted to the surgical department, and empirical intravenous broad-spectrum antibiotic therapy was promptly initiated, along with aggressive fluid resuscitation. Subsequently, an urgent contrast-enhanced CT scan demonstrated a significant fascial defect in the linea alba with herniation of the transverse colon. The colon was surrounded by a massive, multiloculated fluid collection and gas bubbles within the subcutaneous tissue, consistent with an abscess (Figures 2A, 2B). There were no signs of mechanical bowel obstruction or pneumoperitoneum.

**Figure 3:**
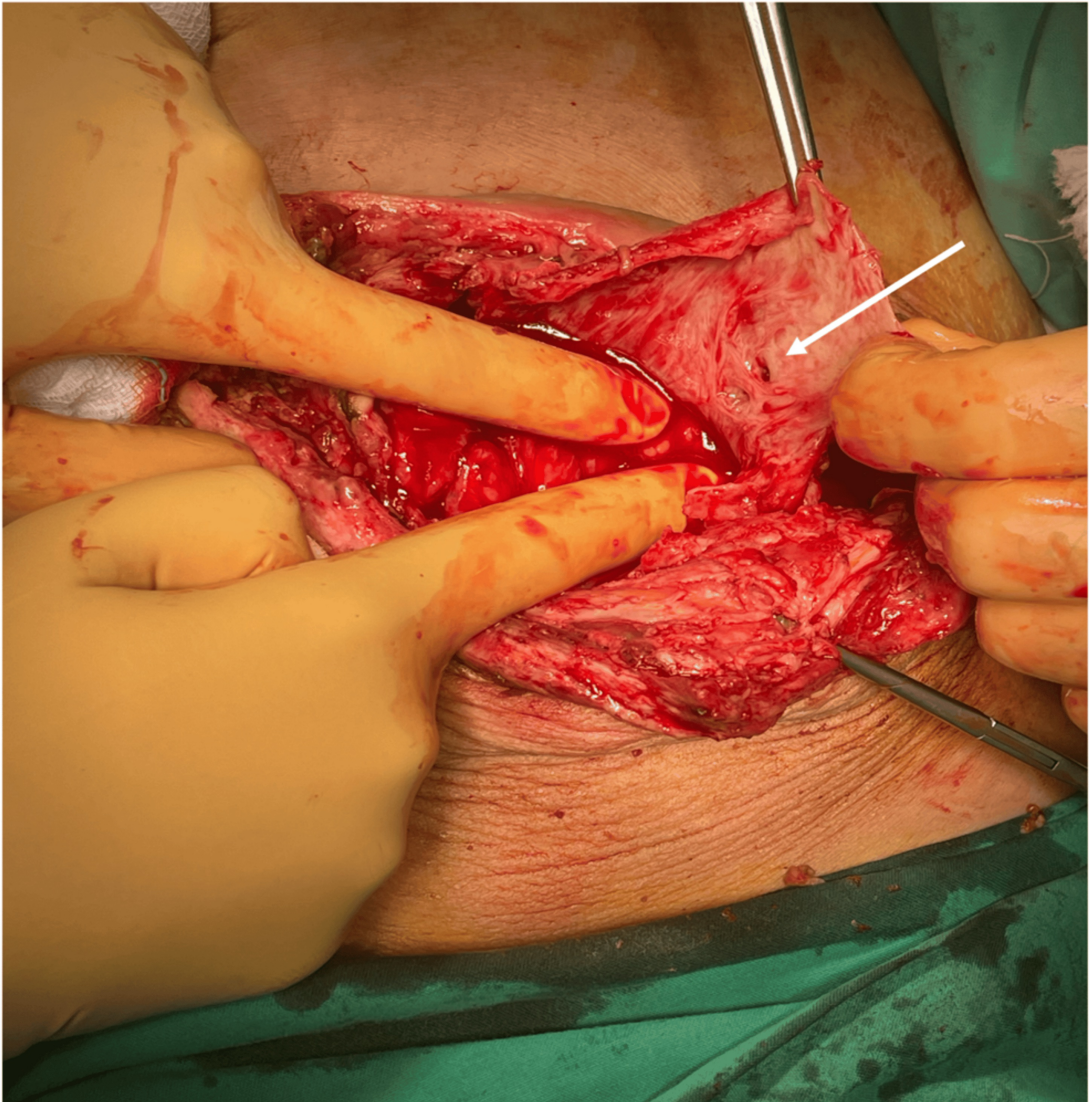
Intraoperative pathological findings. Dense fibrous abscess capsule with clearly visible suspected inflammatory tracts (white arrow) extending from the hernial sac, indicating the pathway of chronic infection into the abdominal wall.

After a detailed discussion regarding the surgical risks and the complexity of the clinical findings, the patient provided written informed consent for the emergency intervention. The patient was taken to the operating room and placed under general anesthesia. An elliptical incision was performed to include the resection of the necrotic overlying skin. Immediately upon entering the subcutaneous space, more than 1 L of foul-smelling purulent material was evacuated using suction, confirming the presence of a massive parietal abscess. Following the evacuation, a dense fibrous abscess capsule was identified and successfully excised (Figure 3).

**Figure 4:**
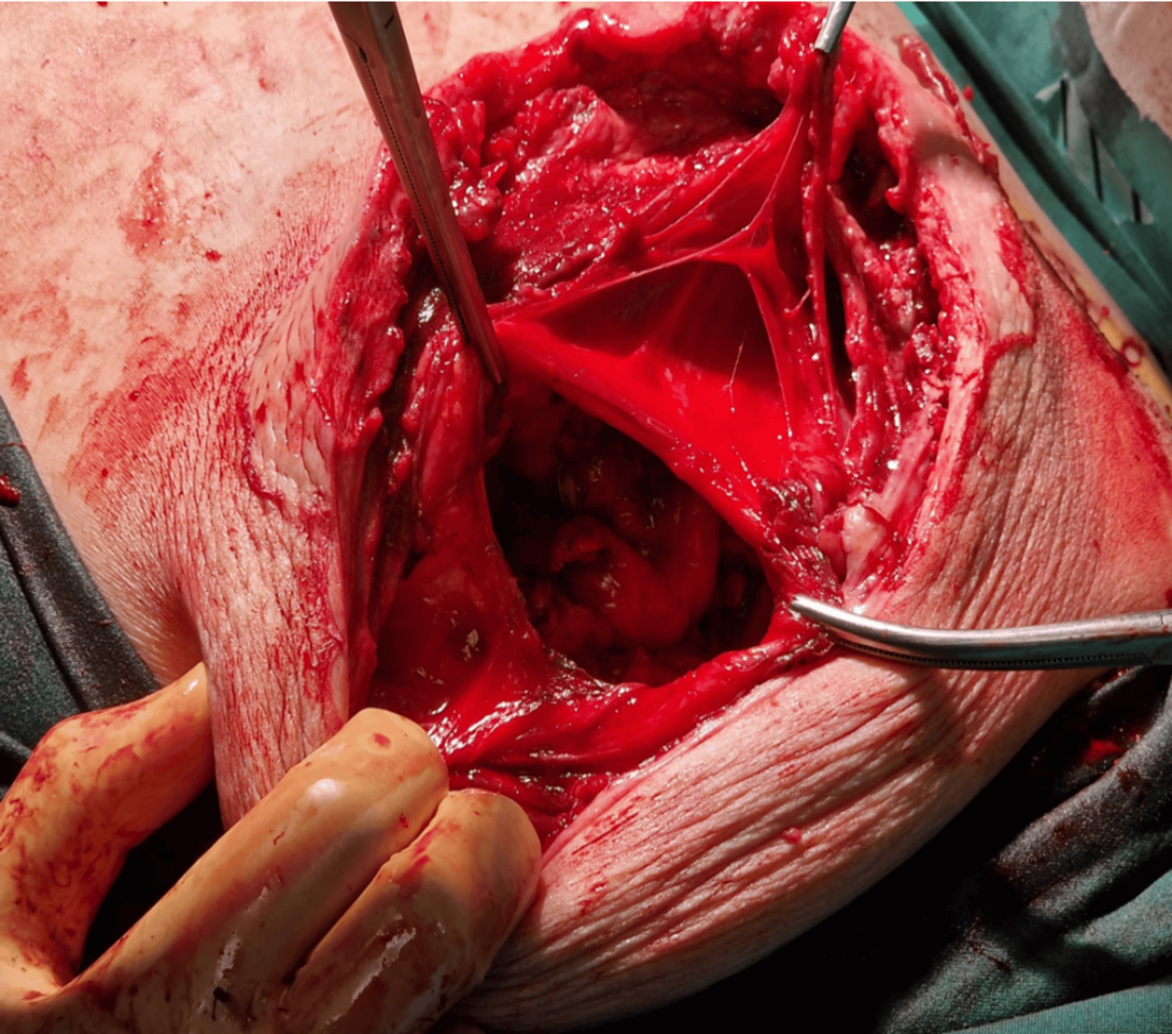
Fascial defect. View of the large (5 cm) fascial defect in the linea alba after the debridement and reduction of the transverse colon.

Upon entering the hernial sac, a significant portion of chronically incarcerated greater omentum was identified; due to its non-viable and chronically inflamed state, an omentectomy was performed using a vessel-sealing energy device. The transverse colon was found within the sac, appearing chronically inflamed but viable, without evident perforation, and was carefully reduced into the peritoneal cavity. Following the reduction, a large fascial defect measuring approximately 5 cm was identified in the linea alba (Figure 4).

Given the contaminated surgical field, prosthetic mesh was avoided. The defect was repaired primarily using a running no. 2 polydioxanone (PDS) loop suture, reinforced with interrupted no. 1 polyester sutures. The surgical site underwent extensive debridement and thorough irrigation with normal saline. Finally, partial skin closure was performed, and a negative pressure wound therapy (NPWT) system was applied to manage the significant dead space created by the abscess evacuation and the skin resection, promote granulation tissue formation, and prevent further infectious complications.

The patient’s early postoperative recovery was uneventful. Culture of the purulent material obtained intraoperatively grew mixed skin and enteric flora. She remained afebrile from the first postoperative day, and inflammatory markers showed a rapid and steady decline. Normal bowel function was restored on the second postoperative day, and the patient was successfully transitioned to a regular diet. She was discharged on postoperative day 5 with the NPWT system in place and instructions for regular outpatient follow-up and dressing changes.

**Figure 5:**
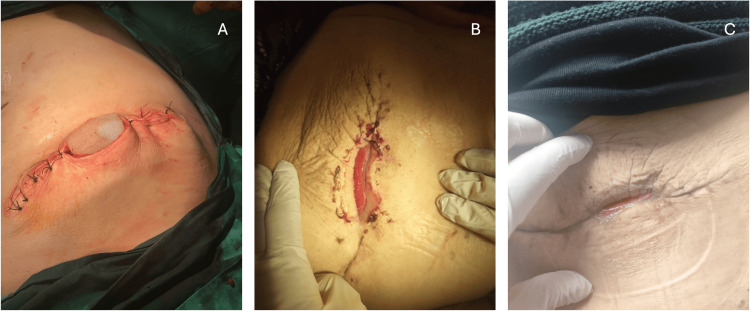
Sequential wound healing under negative pressure wound therapy (NPWT). (A) Partial primary closure of the skin edges and placement of the polyurethane foam dressing within the wound cavity, prior to the initiation of NPWT. (B) Wound appearance on postoperative day 10, showing early granulation tissue. (C) Significant cavity reduction and healthy granulation on day 20.

The NPWT was initially set to the intermittent pressure mode (-125 mmHg/-85 mmHg) to further stimulate granulation tissue formation and optimize local microcirculation. The treatment continued for a total of 21 days. Sequential evaluation of the wound at 10-day intervals demonstrated excellent progression, with the gradual filling of the cavity with healthy granulation tissue and a significant reduction in wound surface area (Figure 5). Following the discontinuation of NPWT, the wound was managed with conventional dressings until complete healing by secondary intention was achieved. At the three-month follow-up, there were no clinical signs of hernia recurrence or late infectious complications. Histopathological evaluation of the resected fibrous capsule and omentum revealed dense fibrocollagenous tissue with intense chronic inflammatory infiltration and areas of fat necrosis, with no evidence of malignancy.

## Discussion

Epigastric hernias, although common in general surgery, are typically small and contain only preperitoneal fat or omentum. The presence of a hollow viscus, specifically the transverse colon, within an epigastric hernia sac is an exceptionally rare clinical entity with few cases having been reported in the literature [5-8]. This rarity is attributed to the anatomical nature of the linea alba defect, which is usually too narrow to allow the protrusion of large bowel loops. However, as demonstrated in our case, chronic, neglected hernias can lead to the gradual enlargement of the fascial defect, eventually permitting the incarceration of the colon [7,8].

Although epigastric hernias containing the colon often present with signs of obstruction, the formation of an extensive abscess, as seen in our case, is independent of luminal patency. The presumed pathophysiology involves bacterial translocation across a compromised colonic wall due to chronic pressure, which likely led to the formation of the abscess even in the absence of a macroscopic perforation [9]. In such instances, the infection may remain localized within the hernial sac, mimicking a primary soft-tissue infection.

CT played a pivotal role in our diagnostic approach, accurately mapping the extent of the multiloculated abscess and the presence of gas bubbles. While the severity of the clinical exam mandates advanced imaging, the diagnostic challenge lies in recognizing that a seemingly localized abdominal wall abscess may be the sentinel sign of a complicated underlying hernia. Without advanced imaging, a simple bedside incision and drainage could have led to catastrophic outcomes, such as unrecognized bowel injury.

The surgical management of contaminated abdominal wall defects remains a subject of debate. The use of prosthetic mesh in a field with gross contamination is generally contraindicated due to the high risk of biofilm formation and chronic infection. We opted for a primary repair reinforced with heavy sutures, a strategy often preferred in emergency settings where infection precludes safe mesh implantation [10].

Finally, the application of NPWT was instrumental in our patient's management. NPWT serves to manage the "dead space" remaining after the evacuation of a massive abscess, reduces local edema, and promotes the formation of healthy granulation tissue [11]. This modality suggests a feasible and effective intervention for improving outcomes in selected complex abdominal wall cases where gross contamination and a high risk of surgical site infection preclude the immediate use of prosthetic materials.

## Conclusions

In conclusion, a massive abdominal wall abscess can be a deceptive clinical presentation of a neglected, long-standing ventral hernia containing transverse colon. Although epigastric hernias are commonly small and contain only preperitoneal fat, the possibility of hollow viscus involvement should always be considered, especially in irreducible cases. This report highlights the critical role of early advanced imaging, such as CT, which is essential for accurate diagnosis, surgical planning, and the prevention of catastrophic intraoperative complications.

Furthermore, our case suggests that primary fascial repair combined with NPWT may be a feasible and effective strategy in selected contaminated cases when source control is achieved and prosthetic mesh is avoided. This approach effectively manages the surgical dead space and promotes granulation tissue formation, providing a viable alternative in complex, infected environments. High clinical suspicion remains the cornerstone for the successful management of these rare and challenging surgical emergencies.
